# The stake of informing patients of the risk of hypofertility after chemotherapy for breast cancer

**DOI:** 10.3389/fpubh.2023.1129198

**Published:** 2023-03-03

**Authors:** Florian Martinet-Kosinski, Sébastien Lamy, Eric Bauvin, Florence Dalenc, Charlotte Vaysse, Pascale Grosclaude

**Affiliations:** ^1^Equity Team: Team Labeled by the French League Against Cancer, UMR1295 CERPOP, Toulouse, France; ^2^Tarn Cancer Registry, Claudius Regaud Institute, Toulouse, France; ^3^Group for Research and Analyses in Public Health (GAP), Claudius Regaud Institute, IUCT-Oncopole, Toulouse, France; ^4^Regional Cancer Network of Occitanie (Onco-Occitanie), Toulouse, France; ^5^Department of Medical Oncology, Claudius Regaud Institute, IUCT-Oncopole, Toulouse, France; ^6^Department of Surgical Oncology, University Hospital Center Toulouse, Institut Universitaire du Cancer de Toulouse-Oncopole, Toulouse, France

**Keywords:** oncofertility, breast cancer, information—access and interaction, social inequalities, woman health

## Abstract

**Introduction:**

Too few women with invasive breast cancer are informed of the risk of hypofertility after chemotherapy. However, this risk can be prevented by offering gamete preservation by a specialized team. We believe that if more women were informed about gamete preservation, more of them would accept it.

**Objectives:**

The primary objective is to describe each step of the oncofertility care pathway from provision of information to gamete preservation. The secondary objective is to estimate the impact of not receiving information by determining the proportion of women who would have undergone gamete preservation if they had been informed.

**Method:**

575 women aged 18–40 years treated with chemotherapy for breast cancer between 2012 and 2017 in the Ouest-Occitanie region (~3 million inhabitants) were included. We first constructed a multivariate predictive model to determine the parameters influencing the uptake of the offer of gamete preservation among women who were informed and then applied it to the population of uninformed women.

**Results:**

Only 39% of women were informed of the risks of hypofertility related to chemotherapy and 11% ultimately received gamete preservation. If all had been informed of the risk, our model predicted an increase in gamete preservation of 15.35% in the youngest women (<30 years), 22.88% in women aged between 30 and 35 years and zero in those aged ≥36 years. We did not find any association with the European Deprivation Index (EDI).

**Conclusion:**

Oncologists should be aware of the need to inform patients aged ≤ 35 years about gamete preservation. If all received such information, the impact in terms of gamete preservation would likely be major.

## 1. Introduction

The preservation of fertility in women with breast cancer receiving anti-cancer treatment is a major public health issue. The problem of fertility reduction by cytotoxics administered for the treatment of non-metastatic invasive breast adenocarcinoma (1) concerns more and more women because an increasing number of them can now hope to be cured and because the age at which women choose pregnancy is increasing, thus increasing the risk of having breast cancer during pregnancy. A social phenomenon is also involved because more and more women are starting a pregnancy late in life after a second union. Since having a child at the age of 35 is no longer rare, it is important to prevent the risk of hypofertility by preserving gametes before the initiation of any chemotherapy. The French public health code stipulates that each patient must be informed of the risks of the therapies they receive. Moreover, since 2004, a law requires gamete preservation to be offered if a treatment is likely to impair fertility (2).

However, recourse to gamete preservation in France is low. In 2018, a study based on national data on cancers diagnosed in 2013 estimated that 10,000 women under the age of 40 were eligible to be informed about the risks for their future fertility due to their treatments and about the available options for gamete preservation (3). From 2013, however, the data on all-cause gamete preservation obtained from medical procreation assistance centers provided by the French National Biomedicine Agency revealed an estimate of about 600 oocytes or samples of ovarian tissue preserved per year (4), increasing to about 2,300 in 2018 (5). These figures are far removed from the potential number of beneficiaries, thus highlighting the need to improve the access to gamete preservation.

During the consultation to propose a suitable therapy to the patient, the oncologist must therefore clearly inform her of the risk of post-chemotherapy hypofertility and offer a consultation with a specialist gynecologist. During the latter, the risks and consequences of anti-cancer treatments on fertility are again discussed, the ovarian reserve of the patient is evaluated and the various options for gamete preservation are presented (6). If the patient's ovarian reserve is sufficient, gamete preservation is offered, and the start of the patient's treatment is delayed accordingly (7).

The pathway leading to gamete preservation therefore requires perfect coordination between the oncology team and the gynecologist specialized in fertility at each step. Most studies until now have described either the transmission of information to patients and access to the oncofertility consultation or the frequency of gamete preservation. Few studies have examined the entire pathway of breast cancer patients from the announcement of the personalized care plan to the actual preservation of gametes in order to assess the attrition of cohorts at the different steps of the pathway.

## 2. Objectives

The main objective of this study is to describe the oncofertility care pathway at each step from providing information to gamete preservation, in a cohort of women with invasive breast cancer representative of the general population. The secondary objective is to estimate the impact of not being informed about this issue by determining the proportion of women who would have decided to benefit from gamete preservation if they had been informed about it.

## 3. Methods and materials

### 3.1. Study population

**Figure 1:**
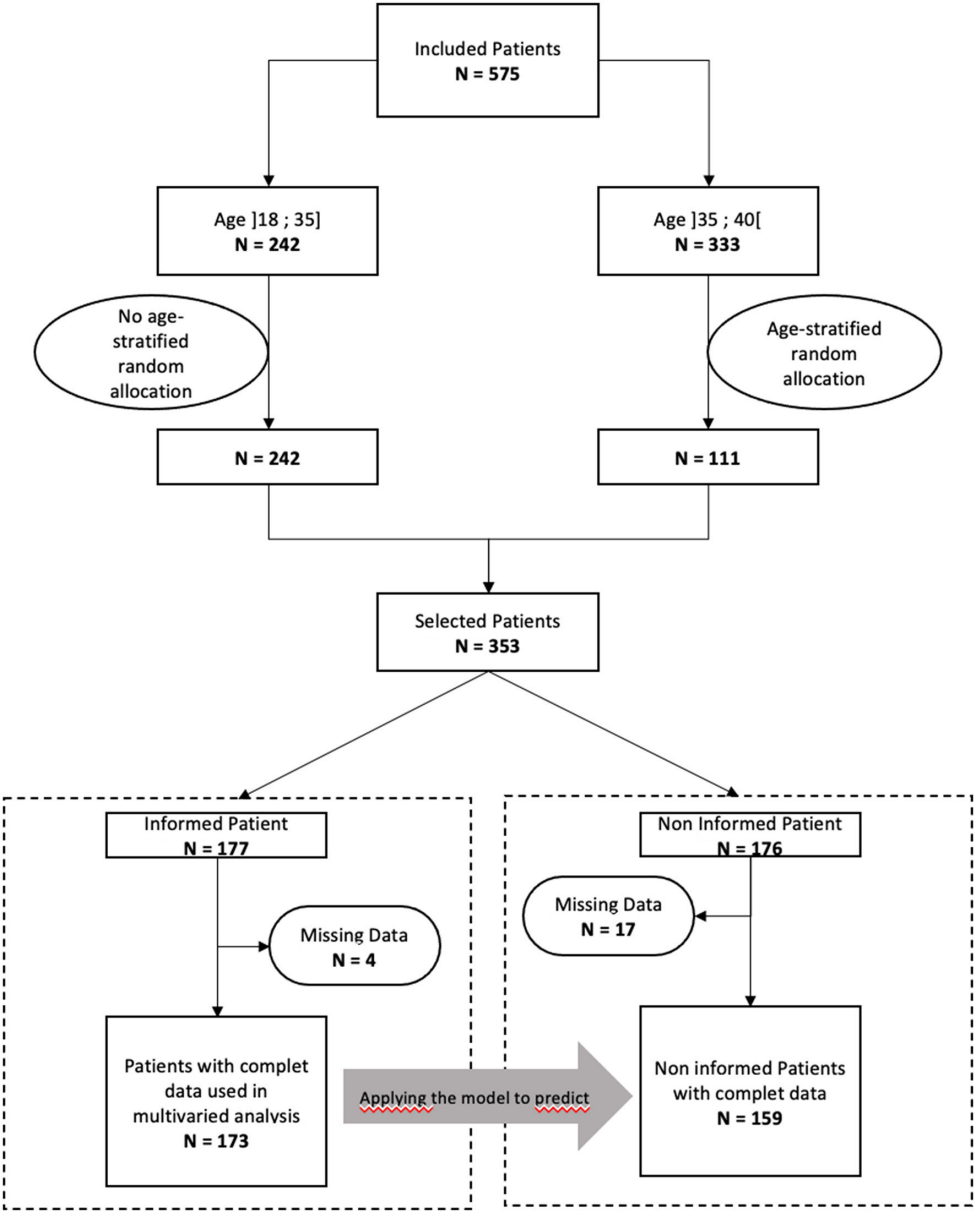
Flowchart.

The study population is a cohort of women aged 18–40 years with infiltrating breast adenocarcinoma eligible for (neo)adjuvant chemotherapy and diagnosed between January 2012 and December 2017 in the Ouest-Occitanie region of France. The cohort was described previously (under review at Scientific Reports). Patients were selected through the regional cancer network's central cancer archive, which collates all patient records discussed in multidisciplinary staff meetings in the region. The administration of chemotherapy was verified in the archive by checking the lists of treatments delivered by the hospital pharmacies, or by contacting the oncology departments directly. Women whose treatment could not be verified were excluded. In order to have a sufficient representation of young women while maintaining a reasonable survey sample size all women aged ≤ 35 years (*n* = 242) were included in the study and one in three women between the ages of 36 and 40 years were selected at random (*n* = 111), resulting in a sample of 353 patients (Table 1 and Figure 1). The same person collected the data from the medical records of all the health centers in the region, whether private or public. Authorization (no. 917235V1) was obtained from the National Commission for Informatics and Liberties (CNIL) to create the database.

**Table 1:** Fertility preservation in the population.

**Variables**		**Total** ^**^		**Fertility preservation**				Chi2^*^
			**No**		**Yes**			
		***N***	**%**	***N***	**%**	***N***	**%**	***P***
		575		513	89.2	62	10.8	
Age	18–29 years	61	10.6	35	57.4	26	42.6	0.0001
	30–35 years	181	31.5	154	85.1	27	14.9	
	36–40 years	333	57.9	324	97.3	9	2.7	
Parity at Diagnosis	0	127	22.1	81	63.8	46	36.2	0.0001
	1	121	21.0	107	88.4	14	11.6	
	2 or more	294	51.1	292	99.3	2	0.7	
	Missing	33	5.7	33	100.0	0	0.0	
Marital status	Not married	117	20.3	94	80.3	23	19.7	0.021
	Married	399	69.4	360	90.2	39	9.8	
	Missing	59	10.3	59	100.0	0	0.0	
EDI	1	133	23.1	117	88.0	16	12.0	0.379
	2	131	22.8	120	91.6	11	8.4	
	3	96	16.7	88	91.7	8	8.3	
	4	105	18.3	94	89.5	11	10.5	
	5	92	16.0	76	82.6	16	17.4	
	Missing	18	3.0	18	100.0	0	0.0	
Initial metastasis	No	512	89.0	454	88.7	58	11.3	0.0715
	Yes	43	7.5	42	97.7	1	2.3	
	Missing	20	3.5	17	85.0	3	15.0	
Neoadjuvant Therapy	No	343	59.7	306	89.2	37	10.8	0.872
	Yes	221	38.4	196	88.7	25	11.3	
	Missing	11	1.9	11	100.0	0	0.0	
Triple negative	No	392	68.2	352	89.8	40	10.2	0.588
	Yes	159	27.6	140	88.1	19	11.9	
	Missing	24	4.2	21	87.5	3	12.5	
Family history	No	413	71.8	372	90.1	41	9.9	0.314
	Yes	110	19.1	95	86.4	15	13.6	
	Missing	52	9.1	46	88.5	6	11.5	
Structures	Teaching hosp	341	59.3	299	87.7	42	12.3	0.525
	Private Tls	154	26.8	139	90.3	15	9.7	
	Private reg	50	8.7	47	94.0	3	6.0	
	Public reg	30	5.2	28	93.3	2	6.7	
Oncologists Gender	Women	315	54.8	281	89.2	34	10.8	0.983
	Men	233	40.5	208	89.3	25	10.7	
	Missing	27	4.7	24	88.9	3	11.1	
Year of diagnosis	2012–2013	211	36.7	207	98.1	4	1.9	0.0001
	2014–2017	364	63.3	306	84.1	58	15.9	

^*^Pearson's Chi2 done on cases with complete data.

^**^353 observations: population size after weighting N = 575.

### 3.2. Variables

#### 3.2.1. Main outcomes

**Figure 2:**
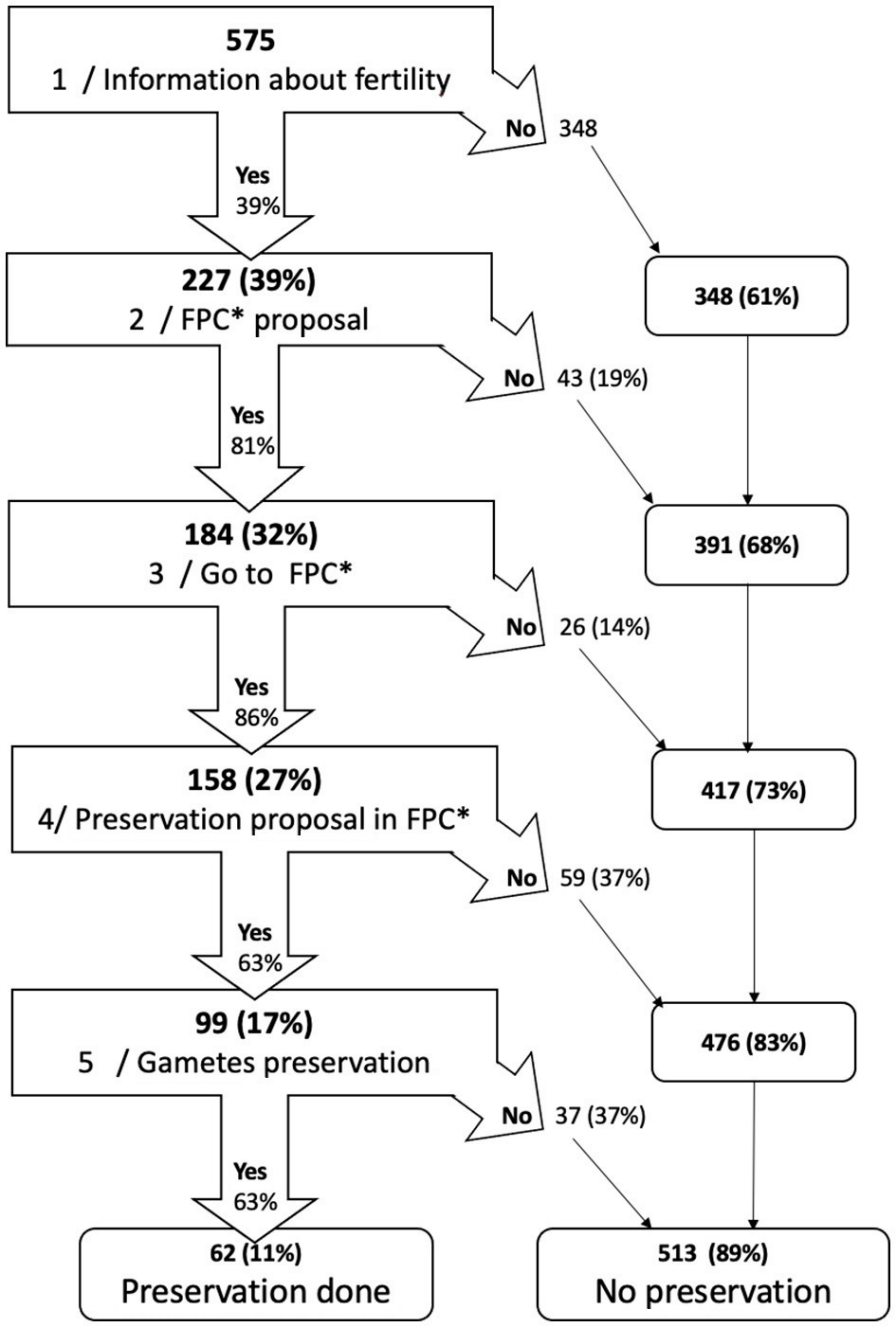
Patient's trajectory to fertility preservation. ^*^FPC, fertility preservation consultation. Bold: cumulative number of cases or percentage, Narrow: conditional number of cases or percentage. Example: step2 FPC proposal: 227 women (39%) were informed of the risks associated with chemotherapy. Fertility presevation consultation was offered to 184 women (81%) and not offerred to 43 (19%). These 43 women were excluded from the process and added to the 348 who had not been informed. At the end of the second step 391 women were excluded (68%).

The gamete preservation pathway comprises the following steps: (1) information on the risk of hypofertility given by the oncologist, (2) offer of consultation with a specialized gynecologist, (3) oncofertility consultation, (4) offer of gamete preservation, depending on the woman's ovarian reserve and not only on her acceptance, and finally, and (5) gamete preservation if the patient so wishes (Figure 2). We sought to establish the proportion of women completing each step of this process.

We considered that a woman had been informed and the consultation offered if this was mentioned in the report of the announcement consultation. For the oncofertility consultation and gamete preservation, we consulted the oncofertility centers of the region whose data were cross-referenced with our cohort. The information on the preservation proposal was found in the oncofertility consultation reports.

#### 3.2.2. Main explanatory variables

We studied the main factors associated with gamete preservation found in the literature:

- Gender-specific factors such as age at diagnosis (divided into three classes: [18–29]; [30–35]; [36–40]), parity (into three classes: 0; 1; >1), marital status at diagnosis, family history of breast cancer (present or not), and social conditions as assessed by the European Deprivation Index (EDI) (8).
- Carcinologic characteristics: year of primary diagnosis (in two time periods: [2012–2013] and [2014–2017]), metastatic status (yes/no), triple-negative tumor status (yes/no), and neoadjuvant chemotherapy administered (yes/no).
- Factors related to the care pathway: type of institution that initiated the medical treatment: university hospital, private hospital in Toulouse, peripheral public hospital, peripheral private hospital, and oncologist (male/female).

### 3.3. Statistical analysis

All analyses were done using STATA software (StataCorp LP, College Station, TX, version 11.1).

#### 3.3.1. Primary objective

We first calculated the proportion of women completing each of the five steps of the pathway. These proportions were obtained in a sample weighted to account for the sampling rate applied to women aged ≥ 36 years (sampling rate: 1/3). The population size was 353 women and increased to 575 women when the study design was considered. All women in the sample were included in this analysis because there was no missing data on the oncofertility care pathway variables. At each step, we present a cumulative percentage across the entire cohort and a percentage calculated from the population of women still involved after the previous step (Figure 2).

#### 3.3.2. Secondary objective

To address our secondary objective, we constructed a multivariate stepwise predictive model.

We built a generalized linear model with a logit link function to predict the completion of gamete preservation among women who were informed. In a first step, we selected associated variables from bivariate analyses. Then, these variables were introduced in a multivariable model. We controlled for the appropriateness of adding each variable using the Akaike information criterion (AIC), which provide a measure of the model log-likelihood penalized by the number of parameters used. The final predictive model was chosen by minimizing the AIC (9) (see Supplementary Appendix for results). The sensitivity and specificity of our final model were checked with a receiver operating characteristic (ROC) curve (ROC curve provided in Supplementary Appendix 2). We then used this model on the population of uninformed women to predict the expected number of women who would have chosen to receive preservation if all had been informed. Given the missing data on the variables considered in the multivariate model, the number of informed women included was 172 (flowchart, Figure 1).

## 4. Results

### 4.1. Description of population

Only 10.8% of all women received gamete preservation. This proportion depended on the patient's age at diagnosis: 42.6% in those < 30 years of age, but only 2.7% in women ≥36 years of age. In addition to age, other factors influencing gamete preservation in this population were the following: parity at diagnosis (fewer than 1% of women with 2 or more children had gamete preservation vs. more than 36% of women without children) and year of primary diagnosis (15.9% in 2014–2017 vs. 1.9 in 2012–2013) (Table 1).

### 4.2. Observed values: Oncofertility pathway

**Figure 3:**
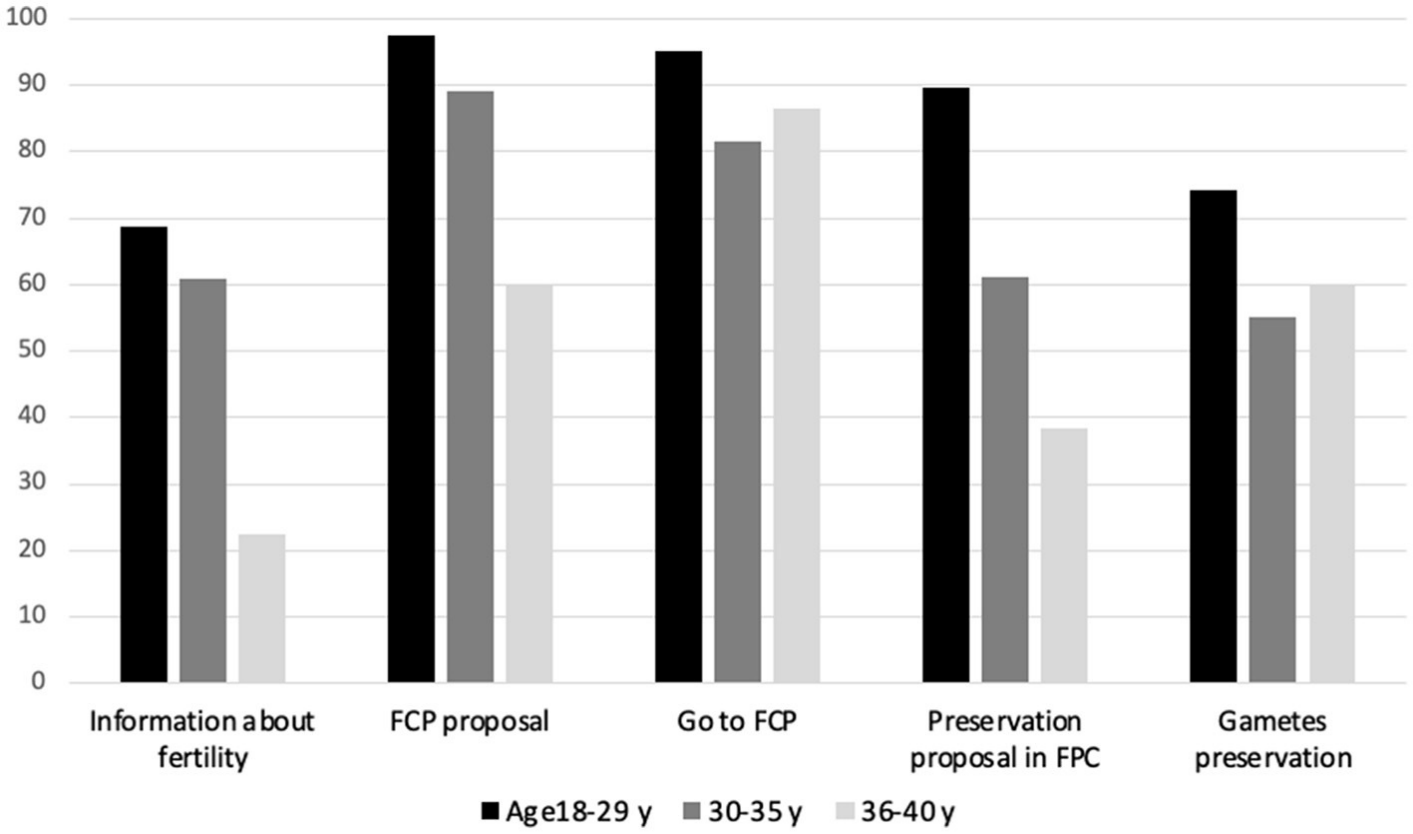
Proportion of positive decisions at different steps of the pathway by age.

Only 39% of women were informed of the risk of hypofertility when they consulted the oncologist. Of those who were informed of the risk, an oncofertility consultation was offered to 81% of them and 86% of them accepted and attended it. Considering all the women in the cohort and not only those who were informed of the risk, only 27% of the patients received an oncofertility consultation. Gamete preservation was offered to 63% of the women who attended an oncofertility consultation, but only 63% of the latter accepted it. Thus, only 10.8% of the entire cohort received gamete preservation (Figure 2). Figure 3 shows the percentage of positive decisions by age group throughout the oncofertility care pathway. It confirms that women aged ≥ 36 years were the least informed.

### 4.3. Predicted values: Preferred model

Our model for predicting gamete preservation on the basis that the subject was informed included the following variables: age and parity at diagnosis, gender of oncologist, and time of diagnosis (Supplementary Appendix 1). This model had a sensitivity of 70.91%, a specificity of 83.05%, a positive predictive value of 66.10% and a negative predictive value of 85.96% (Supplementary Appendix 2) (Table 2).

**Table 2:** Missed opportunity estimation related to the lack of access to initial information.

		**Fertility preservation rate**			
		**(536 women with complete data)** ^**^			
		**Observed**		**Expected if all patients were informed** ^*^	
		**%**	**[95% Conf. interval]**	**%**	**[95% Conf. interval]**
Age	18–29 years	45.6	[33.2; 58.6]	52.6	[39.7; 65.2]
	30–35 years	15.3	[10.6; 21.6]	18.8	[13.6; 25.4]
	36–40 years	1.9	[0.5; 7.4]	1.9	[0.5; 7.4]
Total		10.8	[8.1; 14.3]	12.7	[9.8; 16.3]

^*^Obtained using the prediction of the multivariate model of fertility preservation among informed patients on those who were not informed.

^**^The percentages are slightly different from those in Table 1 because we only use cases with complete data for the variables used in the model.

When applied to the group of women who were not informed, the model predicted that if all patients had been informed, 52.6% of those aged < 30 years and 18.8% of those aged between 30 and 35 years, would have accepted the offer of gamete preservation, whereas no women aged ≥ 36 years would have received it. This represents an increase in gamete preservation of 15.35% in the youngest women (age < 30 years), 22.88% in those of intermediate age (30–35 years), and zero for those with age at diagnosis ≥36 years.

## 5. Discussion

Our study is one of the few to provide a comprehensive view of the oncofertility care pathway in women aged ≤ 40 years with invasive breast cancer, from the transmission of information by the oncologist about the risk of chemo-induced hypofertility to gamete preservation. While the uptake of the offer of gamete preservation increased from 2012 to 2017, thus confirming the results of other studies (10, 11), only 10.8% of women actually received it.

For each step of the oncofertility pathway, we calculated not only a cumulative percentage from the beginning but also a percentage of women who had reached the previous step (Figure 2). The step at which there were the most exclusions was step 1, i.e., information from the oncology team (Figures 2, 3), since only 39% of women were informed of the risk of post-chemotherapy hypofertility. However, our results also show significant exclusions at subsequent steps.

The factors determining the transition from one step to the next are variable. The information given to patients on the subject and the proposal to be referred for an oncofertility consultation obviously depend on the oncologist. Even if the oncologist is legally obligated (2) to provide information and systematically propose a consultation, it appears that this is not done in many cases. There are several possible explanations for this: lack of knowledge of the risks and solutions, lack of time during the consultation, or the misconception that the subject does not need to be mentioned because the patient already has children or is over 35 years of age or has a poor prognosis. In addition, other more subjective factors may be involved (11–13). When a gynecologist offers gamete preservation (step 4), the decision to do so is based mainly on the patient's physiological and biological characteristics (14). Indeed, during the consultation, the specialist gynecologist first studies the patient's ovarian reserve. Only if the latter is sufficient and the benefit-risk ratio positive can gamete preservation be proposed. Women aged ≥ 36 years are much less likely to be offered gamete preservation (Figure 3) because their ovarian reserve is lower owing to their age.

During this process, the patient's choice may be expressed on two occasions: whether to accept the proposed consultation with the specialist gynecologist (step 3) and whether to receive gamete preservation when it is possible (step 5). These choices are obviously influenced by the patient's overall plans for parenthood. In a study conducted between 2018 and 2014 in a population similar to ours, Assi et al. (15) reported that 30% of the 39 women in their small sample intended to become a parent at the time of diagnosis. We were not able to take this fundamental psychosocial factor into account, which constitutes a limitation. Indeed, there was very little information on this subject in the patients' medical files and we did not wish to question them directly about this issue for ethical reasons. Nevertheless, we assume that the intention to become a parent is correlated with the woman's age and parity at the time of diagnosis. However, other proxies of the intention of women with breast cancer to become a parent have been used elsewhere, such as marital status. For example, in a study published in 2014 by Ruddy et al. (15) concerning women < 45 years of age with breast cancer, 37% of those younger than 40 years of age intended before their diagnosis to give birth to a child. They also reported that married women were more concerned about the risk of post-treatment hypofertility than other women (16). A similar study by Ruggeri et al. (17) also showed that married women were more concerned about the risk of hypofertility than single women. In our study, marital status did not appear to be correlated with receiving gamete preservation after adjustment for age and number of children. Furthermore, patients' choices may also depend on other more subjective factors such as the way in which the risks of hypofertility are presented to them, the prognosis of their disease, and their belief in being able to become pregnant after treatment. Another limitation of our study is that we were not able to investigate the nature of the information that the women received and the way in which it was transmitted.

We found (Figure 3) an age gradient for the successive steps of the process where the woman's choice is not expressed (i.e., transmission of information, proposal for consultation and proposal for preservation): the older the women were, the less they were informed or offered interventions. On the other hand, when women were able to express themselves because they were informed, this age gradient disappeared for those over 30 years of age. The first two steps (information and consultation) differ from the offer of gamete preservation in that they do not depend on the technical feasibility of preservation. The age gradient observed in these first two steps may be explained by an *a priori* selection made by the oncologists on the basis of the age and/or parity of their patients. Findings on the uptake of the offer of a specialist consultation and gamete preservation, i.e., steps where women's choice is expressed, suggest that this selection was greater in women over 35 years of age, since they were proportionally more numerous in participating in the following steps.

In the PREFER study conducted in Italy between 2012 and 2020 evaluating the reasons for acceptance or refusal of gamete preservation in patients with breast cancer, Blondeaux et al. (18) showed that although 95% of 159 women aged ≤ 40 years were concerned about the problem of post-chemotherapy hypofertility after receiving information, only 34% accepted the offer of an oncofertility consultation. In our study, 27% of women accepted it, but this percentage increased to 69% (=158/227) when only informed women were considered. This difference is probably due to the fact that the PREFER study was an interventional study in which all women received systematic and standardized information, whereas in our observational study, information was probably given mainly to the youngest and/or most motivated women and varied according to the doctors giving it and/or the health centers in which it was given.

In a series of 149 women aged 18–39 years who received chemotherapy or radiation therapy between 2000 and 2012, Yee et al. showed that 78% received information about fertility, 30% had a consultation with a fertility specialist, and 11% accepted gamete preservation (19). In our study, which focused on women who underwent chemotherapy and at a slightly later step, we found that apart from the proportion of women who received information, which was lower (39%), the rates of women who had a consultation (27%) and gamete preservation (11%) were similar. In a retrospective series in the USA between 2006 and 2014, McCray et al. (20) reported that of 303 patients aged 40 years and under, 80 (26%) had a fertility discussion with a physician. Of these, 55 (18% of the total sample) had a fertility consultation. In our series, 39% of women had a fertility discussion and 27% had a fertility consultation. In the American study, among the 55 patients who had a consultation, 17 (or 5.6% of the total number) received gamete preservation, as opposed to 11% in our series. Unlike us, however, the American study also included the results of women who received potential protection by a GnRH agonist, alone or in addition to an oncofertility consultation.

Providing appropriate information is an essential step toward potential gamete preservation. In the FEERIC study, a recent study who is a web-based cohort study launched with a French collaborative research platform, 29% of women reported not undergoing gamete preservation because this option was not offered. Of the 517 women involved in this study aged 40 or younger, 72.4% recalled being informed about the consequences of the treatments on fertility. Specialized oncofertility counseling was offered to 45.6%, it was performed in 39.7 and 24.0% underwent gamete preservation. This study shows that gamete preservation is three times more common in women with a high level of education and the women who participated in this study have a much higher level of education than the population (88% have a university degree) (21). In fact, the low level of information provision that we observed in our patients must be improved in line with French legislation, given that it does not depend on any bio-clinical constraint. With this in mind, we developed a model predicting the probability of gamete preservation in the case where all women are informed. The model forecast a maximum proportion of women benefiting from gamete preservation of 12.7%, i.e., an absolute increase of 1.9 percentage points and a relative increase of 17% (Table 2). If we consider only the youngest women under 30 years of age and those between 30 and 35 years of age, the relative increase would have been 15 and 22% respectively if they had been informed. In contrast, the model did not predict an increase in preservation among women aged 36 and over. This result highlights the importance of targeting women aged 35 and under when providing information, without forgetting older women. The model is statistically satisfactory, with a high sensitivity and specificity (Supplementary Appendix 2). However, we constructed the model on one population (informed women) and then applied it to another (uninformed women), which may have differed from the first in terms of characteristics that we did not include in our study, yet the model assumes that the information was not associated with characteristics that were not studied. Our choice of variables to build the model was based on data in the literature and the possibility of collecting them. Other parameters might therefore explain the choice of preservation with more precision, e.g., the desire to become a parent or the quality of the information given.

We may also have underestimated the proportion of women informed. In fact, it was sometimes difficult to trace the transmission of information by the oncologist. When this information was noted down in the patients' files, we assumed that the doctor had discussed eventual fertility problems related to chemotherapy. However, information may have been given but not recorded as such, either because the physician forgot to report it in the consultation report or because it was recorded in another document to which we did not have access. Nevertheless, unlike the information given to the patient, the existence of a consultation could be ascertained since we cross-referenced our files with those of the only two fertility centers in the region.

Another limitation is that our study concerns only one region, while recommendations concerning the oncofertility care pathway laid down in the French Cancer Plans apply nationally (22). However, our findings are likely generalizable to the rest of France since oncofertility is one of the themes of this national cancer plan and is approached similarly nationwide.

## 6. Conclusion

In this population-based study of women with invasive breast cancer, gamete preservation was performed in only 11%. This inadequate rate is mainly due to the low percentage of women who were informed about it (39%). Providing appropriate information is an important step in the oncology and oncofertility pathway, even though it is not the only explanation for the lack of recourse to preservation. Patients have the right to receive information, a sine qua non condition for respecting their autonomy (23). It is incumbent on oncology care teams to provide information, and they must be better trained to do so. Informing all women under 35 years of age would have a major impact. Women aged 36–40 years should also receive such information, even though it does not seem to increase uptake of gamete preservation in this age group. We should perhaps rethink the age cutoff points since the average age at childbearing increased steadily from 29.3 years in 1999 to 30.7 years in 2019. The age of first pregnancy is increasing, as is the fertility rate of women over 35: from 4.8 in 1999 to 7% in 2019 for women aged 35–39 and from 0.5 in 1999 to 0.9% in 2019 for women aged 40–50 (24).

The decision whether to embark upon the oncofertility care pathway must be made *via* a caring explanatory discussion between the physician and the patient. The patient must be allowed to express her wishes and expectations (desire for pregnancy, benefit/risk ratio) so that the final decision is acceptable and accepted by both the patient and the physician, as expressed by Habermas in his analysis of the ethics of discussion (25). While the amount of information transmitted can be objectively quantified, it is the quality of that information and the way in which it is understood by the patient that should take of place. Our results therefore call for further reflection on these ethical issues. Indeed, our results lead us to reflect more deeply on issues such as health information, free will, utilitarianism etc.

## Data availability statement

The raw data supporting the conclusions of this article will be made available by the authors, without undue reservation.

## Author contributions

FM-K: data collection, analysis, writing, and proofreading. SL: research design, analysis, writing, and proofreading. EB, FD, and CV: data collection and proofreading. PG: research design, data collection, analysis, writing, and proofreading. All authors contributed to the article and approved the submitted version.
