# Supplementary material for: The stake of informing patients of the risk of hypofertility after chemotherapy for breast cancer
Source: Front Public Health. 2023 Mar 3;11:1129198. doi: 10.3389/fpubh.2023.1129198 (PMC10027074; doi:10.3389/fpubh.2023.1129198)
Supplement: Supplementary file 1 [file Table_1.pdf]

		OR	[95% Conf.	Interval]	P>z
age	18-29 y	1*			
	30-35 y	0.35	[0.14 ;	0.88]	0.026
	36-40 y	0.11	[0.02 ;	0.65]	0.015
parity at diagnosis	0	1*			
	1	0.34	[0.14 ;	0.84]	0.019
	2 or more	0.05	[0.01 ;	0.22]	0.000
Oncologists gender	Women	1*			
	Men	2.70	[1.08 ;	6.77]	0.034
year of diagnosis	2011-2013	1*			
	2014-2017	14.51	[4.00 ;	52.64]	0.000
	_cons	0.21	[0.06 ;	0.79]	0.022
Model Obs		N=173			
Log likelihood (null)		-108.1753			
Log likelihood (model)		-69.88572			
degree of freedom		7			
Akaike information criterion		153.7714			
BIC		175.8445			

* reference category

Appendix 1: Multivariate model - Access to fertility preservation according to patient and oncologist characteristics in patients who have been informed.
