# Supplementary figures and images for: The stake of informing patients of the risk of hypofertility after chemotherapy for breast cancer
Source: Front Public Health. 2023 Mar 3;11:1129198. doi: 10.3389/fpubh.2023.1129198 (PMC10027074; doi:10.3389/fpubh.2023.1129198)

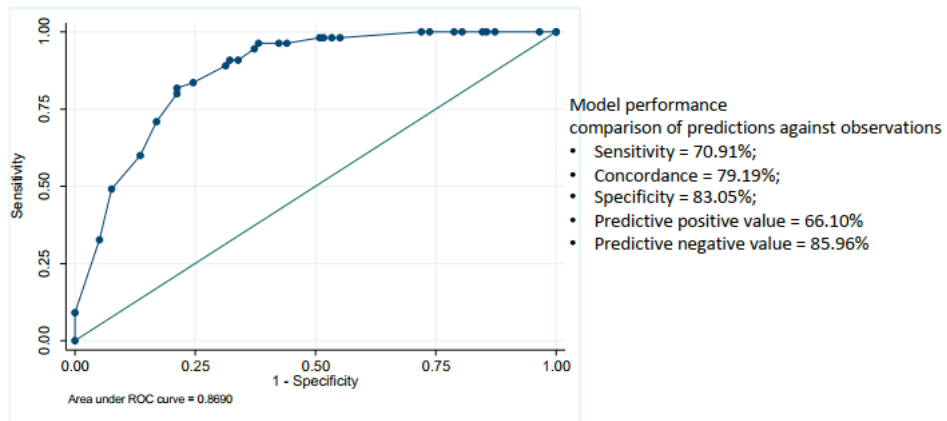

Appendix 2: Model's ROC curve and model performance

Supplement: Supplementary file 2 [file Table_2.pdf]
